# Natural history of large adrenal tumors

**DOI:** 10.3389/fendo.2026.1774131

**Published:** 2026-02-18

**Authors:** Magdalena Macech, Maciej Stępień, Joanna Podgórska, Sadegh Toutounchi, Łukasz Koperski, Joanna Barankiewicz, Małgorzata Bobrowicz, Agnieszka Kondracka, Urszula Ambroziak

**Affiliations:** 1 Department of Internal Medicine and Endocrinology, Medical University of Warsaw, Warsaw, Poland; 2 Second Department of Clinical Radiology, Medical University of Warsaw, Warsaw, Poland; 3 Department of General, Vascular, Endocrine and Transplant Surgery, Medical University of Warsaw, Warsaw, Poland; 4 Department of Pathology, Medical University of Warsaw, Warsaw, Poland; 5 Laboratory of Cellular and Genetic Therapies, Center for Preclinical Research, Medical University of Warsaw, Warsaw, Poland

**Keywords:** adrenal imaging, adrenal incidentaloma, cortisol secretion, follow-up, large adrenal tumor, mild autonomous cortisol secretion (MACS)

## Abstract

**Objective:**

The aim of this study was to characterize large adrenal tumors (LATs), assess the diagnostic accuracy of imaging modalities in detecting malignant LATs, and determine whether further follow-up of seemingly benign LATs is necessary.

**Patients and methods:**

We retrospectively analyzed the clinical, biochemical, and imaging characteristics at presentation and during follow-up in a single-center cohort of patients with adrenal tumors ≥4 cm in diameter evaluated between June 2013 and June 2023.

**Results:**

Of 1,473 patients with adrenal tumors, 269 (18.3%) had lesions measuring ≥4 cm in diameter, of whom 220 were included in the study. Among LATs, 34.6% were adrenocortical adenomas, 27.3% other benign adrenal tumors, 17.3% adrenocortical carcinomas, 11.4% other malignant tumors, and 9.6% pheochromocytomas. Overall, 44.7% of tumors were non-functioning, while 37.8% secreted corticosteroids. Sensitivity of unenhanced computed tomography attenuation values in detecting malignancy was 100% using the threshold >10 Hounsfield units (HU) and 89.5% for >20 HU (with specificity 66.1% and 81.4%, respectively). Magnetic resonance with chemical shift analysis demonstrated high sensitivity (94.4%) but low specificity (56.8%). ^18^F-fluorodeoxyglucose-positron emission tomography integrated with computed tomography showed 100% sensitivity using an adrenal liver ratio > 1. When the maximum standardized uptake value >5 cutoff was applied, sensitivity remained 100%, while specificity improved (100% vs. 82.1%). Eighty-seven tumors were managed with follow-up, with a mean duration of 30.1 months. Significant tumor growth occurred in four cases (4.6%): three myelolipomas and one indeterminate lesion in a patient with extra-adrenal malignancy. Adrenalectomy was performed in six patients (6.9%). Although these tumors were initially classified as adrenocortical adenomas, histopathology revealed benign lesions in five cases and adrenocortical carcinoma in one. The risk of developing mild autonomous cortisol secretion among non-functioning tumors was 6.7%. No progression to overt Cushing’s syndrome was observed.

**Conclusion:**

Although the risk of malignancy increases with tumor size, most LATs are benign. Imaging modalities demonstrate high sensitivity for detecting malignancy in LATs. No malignancies were identified in homogeneous lesions with attenuation <10 HU. LATs that do not meet these benign imaging criteria should undergo multidisciplinary evaluation and continued follow-up.

## 1 Introduction

Adrenal tumors are detected in approximately 1.9%–5% of patients undergoing cross-sectional imaging (1–4). However, population-based studies estimate their prevalence at around 0.5% (5). Most adrenal tumors are discovered incidentally and represent non-functioning, benign adrenocortical lesions that do not require adrenalectomy or further follow-up (6). Large adrenal tumors (LATs) are defined as lesions measuring >4–6 cm and account for approximately 8.6%–38.6% of all adrenal tumors (7, 8). Although the risk of malignancy is higher in tumors >4 cm, the majority of LATs remain benign. The most common diagnoses include adrenocortical adenomas (31%) and pheochromocytomas (22%), with other benign lesions accounting for approximately 16%. Adrenocortical carcinomas represent 13% of LATs, while other malignant lesions constitute 18% (8).

In each case of an adrenal tumor, assessment of malignancy risk is essential. The risk is primarily determined by imaging characteristics and tumor size (6). A tumor size threshold of 4 cm is associated with a sensitivity of 80%–93% for detecting malignant masses but is characterized by low specificity—34%–61% (9–11). According to large meta-analyses, the gold standard initial imaging modality for differentiating benign from malignant adrenal tumors is unenhanced computed tomography (CT), which provides the information on the lipid content. An attenuation value >10 Hounsfield units (HU) demonstrates 100% sensitivity and 57.5% specificity for malignancy, whereas a threshold >20 HU provides comparable sensitivity with a higher specificity (approximately 76%) (6). Consequently, current guidelines suggest the use of these two thresholds for risk stratification. In LATs, the diagnostic accuracy of unenhanced CT for identifying malignant lesions remains similarly high (8). In cases of indeterminate tumors (attenuation 11–20 HU and homogeneous or heterogeneous), additional imaging may be useful to further assess malignant potential. The current options include magnetic resonance imaging (MRI), contrast-enhanced CT, or ^18^F-fluorodeoxyglucose positron emission tomography combined with CT (^18^F-FDG PET/CT). Otherwise, surgical management should be promptly considered (6). Lipid content on MRI is assessed by evaluating signal intensity loss on out-of-phase images using chemical shift analysis. MRI chemical shift imaging demonstrates a sensitivity of approximately 86%–90% and a specificity of 85% in detecting malignant masses (12).

Contrast-enhanced CT allows assessment of contrast washout, reflecting differences in vascularization between benign and malignant lesions. An absolute washout >60% or a relative washout >40% suggests benign pathology (13–15). Among patients without a history of extra-adrenal malignancy, the sensitivity of contrast-enhanced CT for detecting malignancy is 93%–100%, along with a specificity of 92%–100% (12). However, in patients undergoing cancer staging imaging for an extra-adrenal primary malignancy, sensitivity decreases to 16%, while specificity remains high (95%) (12). A more recent study proposed a higher relative washout threshold of 58%, yielding 100% sensitivity but low specificity (16).

^18^F-FDG PET/CT differentiates malignant and benign lesions based on glucose metabolism, which correlates with cellular metabolic activity. In a large retrospective study comprising more than 300 patients, both the sensitivity and specificity of an adrenal liver ratio of more than 1.8 were 86%, whereas the use of a maximum standardized uptake value (SUV max) >4.5 had a sensitivity of 89% and specificity of 76% (17). When imaging is inconclusive and a lesion remains indeterminate, further management strategies, including adrenalectomy or interval imaging, should be discussed within a multidisciplinary team.

Numerous guidelines addressing the management of adrenal incidentalomas have been published, proposing different follow-up strategies based on tumor size, imaging characteristics, and hormonal activity (18–24). Recent guidelines from the European Society of Endocrinology (ESE) and the European Network for the Study of Adrenal Tumors (ENSAT) recommend against further imaging follow-up in patients with an adrenal lesion exhibiting clear benign features (homogeneous appearance and attenuation ≤10 HU) regardless of tumor size (6). This represents an update from the previous guideline from 2016, which suggested no further follow-up for tumors <4 cm with benign imaging features (18).

For several decades, adrenal lesions larger than 4 cm were surgically removed based mainly on their size, regardless of imaging characteristics. This approach has resulted in limited data regarding the natural history of LATs and has contributed to ongoing uncertainty regarding their optimal management, highlighting the need for individualized management strategies (25).

The aim of this study was to characterize LATs, assess the diagnostic accuracy of imaging modalities in detecting malignant LATs, and determine whether further follow-up of seemingly benign LATs is necessary in a retrospective, single-center cohort of patients with adrenal tumors ≥4 cm in diameter evaluated over a 10-year period.

## 2 Patients and methods

### 2.1 Study design

This was a retrospective cohort study conducted at the Department of Internal Medicine and Endocrinology, Medical University of Warsaw, Poland, between June 2013 and June 2023. The study was approved by the local Ethics Committee of the Medical University of Warsaw.

The inclusion criteria were as follows: (1) age ≥18 years; (2) tumor size ≥4 cm measured at the maximum diameter; and (3) tumor classification established by histopathology or by at least two imaging scans performed at least six months apart (except for metastatic lesions and non-operative ACC).

Tumors were categorized into five groups: pheochromocytomas (PHEO), adrenocortical adenomas (ACA), other benign tumors (OB), adrenocortical carcinomas (ACC), and other malignant tumors (OM). Overt hypercortisolism, primary hyperaldosteronism, and catecholamine excess were diagnosed according to the most recent guidelines (26–28). Mild autonomous cortisol secretion (MACS) was defined as a serum cortisol level >1.8 μg/dl after the 1-mg dexamethasone suppression test (6).

Subsequently, tumor characteristics, including size, homogeneity, and density assessed by CT or MRI chemical shift imaging, were evaluated on baseline and follow-up scans in patients who did not undergo adrenalectomy.

### 2.2 Statistical analyses

Statistical analyses were performed using STATA software (ver. 18.5). The findings of the descriptive analysis were presented as frequencies (percentages) for categorical variables and medians (ranges) for continuous variables. The normality of data distribution was verified with the Shapiro–Wilk test. For normally distributed continuous variables, comparisons between two groups were performed using the t-test; for non-normally distributed data, the Mann–Whitney *U* test was applied. Comparisons across more than two groups were carried out using the Kruskal–Wallis’s test. Categorical variables were analyzed using Pearson’s χ² test or Fisher’s exact test when expected cell frequencies were small. When multiple comparisons were performed, the Bonferroni correction was applied. A two-tailed *p*-value < 0.05 was considered statistically significant.

## 3 Results

### 3.1 Patients

**Figure 1:**
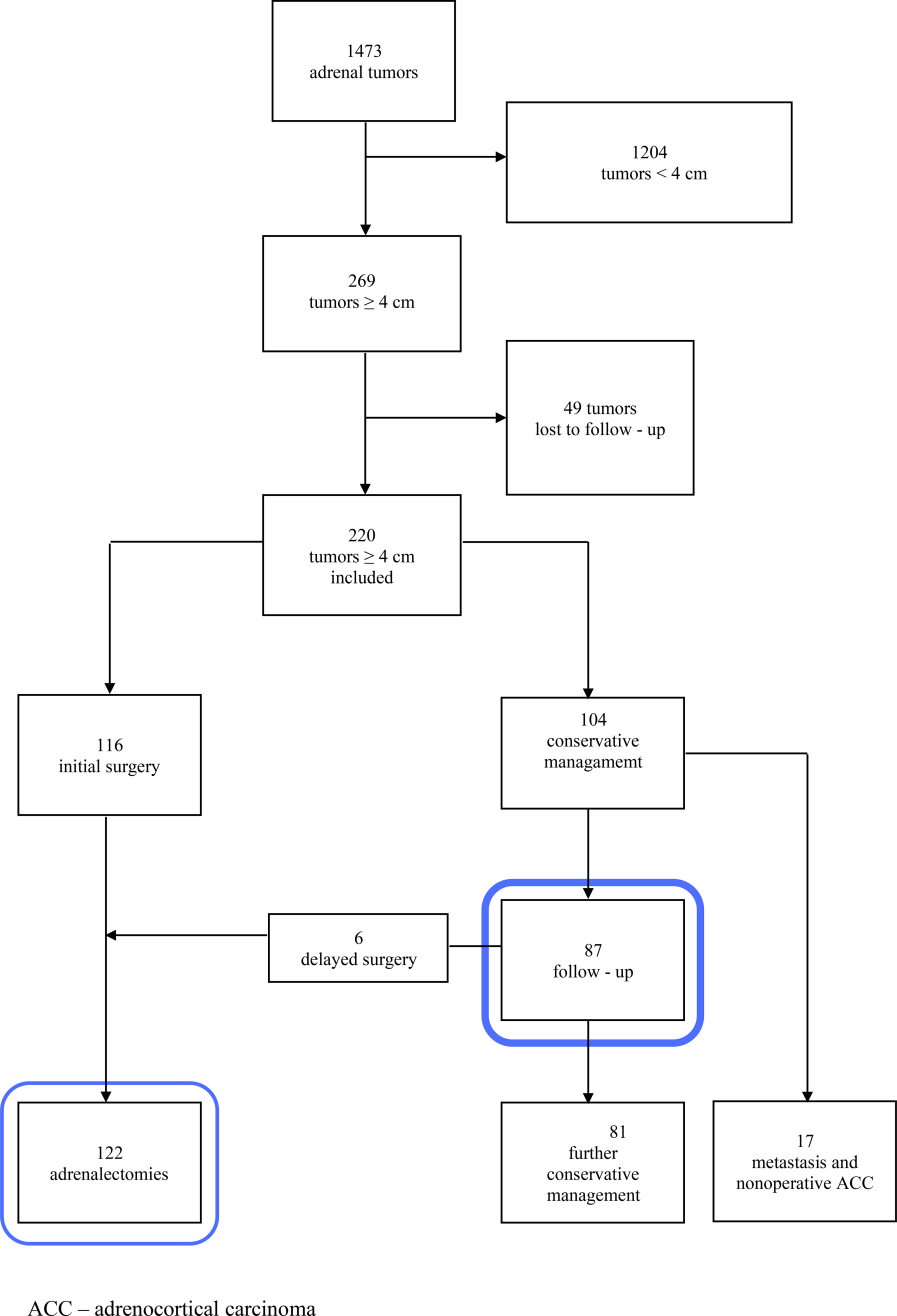
Flowchart of patient inclusion and management.

During the 10-year study period, 1,473 patients with adrenal tumors were evaluated. Among the 269 (18.3%) tumors measuring more than 4 cm, 220 were included in the study, while 49 patients were lost to follow up. Immediate adrenalectomy was performed in 116 patients, whereas 87 were initially managed conservatively. During follow-up, 6 patients were subsequently referred to adrenalectomy, while 81 remained under conservative management (**Figure 1**).

Among LATs, adrenocortical adenoma was the most frequently diagnosed lesion (34.6%). In the ACA group, the median tumor size was 45.00 mm (range: 40–86 mm), and the median unenhanced attenuation was 2.5 HU (range: −20 to 40 HU). Hormonal activity was observed in 77.6% of adenomas, with mild autonomous cortisol secretion being the most common condition (72.4%). Cushing’s syndrome was identified in 2.6% of cases. OB accounted for 27.3% of adrenal tumors. The median tumor size was 55.5 mm (range: 40–150 mm) with a median attenuation of 15.0 HU (range: −87 to 60 HU). Malignant lesions accounted for 28.7% of LATs, including ACC (17.3%) and OM (11.4%). The median tumor size among ACCs was 95.0 mm (range: 40–241 mm), and the median attenuation was 35.0 HU (range: 17–50 HU). The majority of ACCs were hormonally active (77.2%), most commonly secreting cortisol (34.3%). Cushing’s syndrome was diagnosed in 25.7% of patients with ACC (**Table 1**). Within the group of OM, lung cancer metastases were most frequently identified (32%), followed by renal cancer metastases (16%). The remaining lesions comprised a heterogeneous group of malignant neoplasms, including metastases from other or unknown primary origins, sarcomas, and angiosarcomas.

**Table 1:** Demographic characteristics, imaging, and biochemical presentation of patients with large tumors.

Variables	PHEO (*n* = 21, 9.6%)	ACA (*n* = 76, 34.6%)	OB (*n* = 60, 27.3%)	ACC (*n* = 38, 17.3%)	OM (*n* = 25, 11.4%)	TOTAL *n* = 220 (100%)	*P* (overall)
Age (*y*)							**< 0.001**
Median (Min. – max.)	44.0; (19–71)	62.0;(31–78)	60.0; (20–86)	55.0; (18–74)	64.0; (37–89)	59.5; (18–89)	
Sex: female, *n* (%)	15 (74.4)	50 (65.8)	33 (55.0)	26 (68.4)	12 (48.0)	136 (61.8)	0.264
Tumor diameter (mm), *n* (%)							**< 0.001**
Median ±SD Min. – max.	60.0 ± 18.9 40–114	45.0 ± 7.6 40–86	55.5 ± 24.8 40–150	95.0 ± 51.6 40–241	67.0 ± 24.4 41–130	2.0 ± 33.2 40–241	
Tumor density (HU)							**< 0.001**
Median ± SD Min. – max. Available in *n* (%)	30.0 ± 7.4 20–48 11 (52.4)	2.5 ± 12.4 −20–40 74 (97.4)	15.0 ± 47.5 −87–60 44 (73.3)	35.0 ± 6.8 17–50 25 (65.8)	25.0 ± 18.9 15– 90 13 (52.0)	15.0 ± 27.6 −87–90 167 (75.9)	
Hormonal activity							**< 0.001**
No hormonal excess Hormonal excess Cortisol excess CS MACS Hyperaldosteronism Androgen excess Mixed Metanephrine excess Available in *n* (%)	0 21(100) 0 0 0 0 0 0 21 (100%) 21 (100)	17 (22.4) 59 (77.6) 57 (75.0) 2 (2.6) 55 (72.4) 0 0 2 (2.6) 0 76 (100)	52 (86.7) 8 (13.3) 8 (13.3) 0 8 (13.3) 0 0 0 0 60 (100)	8 (22.8) 27 (77.2) 12 (34.3) 9 (25.7) 3 (8.6) 0 4 (11.4) 11 (31.4) 0 35 (92.1)	20 (80.0) 5 (20.0) 5 (20.0) 0 5 (20.0) 0 0 0 0 25 (100)	97 (44.7) 120 (55.3) 82 (37.8) 11 (5.1) 71 (32.7) 0 4 (1.8) 13 (6.0) 21 (9.7) 217 (98.6)	
Management, *n* (%)							**< 0.001**
Conservative Surgical	21 (100)	44 (57.9) 32 (42.1)	34 (56.7) 26 (43.3)	4 (10.5) 34 (89.5)	16 (64) 9 (36)	98 (44.5) 122 (55.5)	
Mode of discovery, *n* (%)							**< 0.001**
Incidental Hormone excess Pain Other	11 (52.4) 6 (28.6) 1 (4.8) 3 (14.3)	65 (85.5) 0 5 (6.6) 6 (7.9)	43 (71.3) 0 12 (20) 5 (8.3)	10 (26.3) 10 (26.3) 13 (34.2) 5 (13.2)	13 (52.0) 0 6 (24.0) 6 (24.0)	142 (64.5) 16 (7.3) 37 (16.8) 25 (11.4)	
Location of adrenal tumor, *n* (%)							0.175
Right Left Bilateral	11 (52.4) 10 (47.6) 0	32 (42.1) 35 (46.1) 9 (11.8)	28 (46.7) 27 (45.0) 5 (8.3)	15 (39.5) 23 (60.5) 0	14 (56.0) 8 (32.0) 3 (12.0)	100 (45.5) 103 (46.8) 17 (7.7)	

PHEO, pheochromocytoma; ACA, adrenocortical adenoma; OB, other benign lesion; ACC, adrenocortical carcinoma; OM, other malignant lesion; n, number; SD, standard deviation; HU, Hounsfield units; CS, Cushing syndrome; MACS, mild autonomous cortisol secretion.

Bold values indicate statistically significant differences (p < 0.05).

Pheochromocytomas accounted for 9.6% of LATs. All PHEOs demonstrated attenuation values >10 HU on unenhanced CT and were confirmed by preoperative biochemical testing.

### 3.2 Benign and malignant tumors

Compared with benign lesions, malignant lesions were significantly larger (47.0 vs. 75.5 mm, *p* < 0.001) and demonstrated higher attenuation on unenhanced CT (4.0 vs. 33 HU, *p* < 0.001). Benign tumors were more frequently detected incidentally (79.4% vs. 36.5%, *p* < 0.001), whereas malignant lesions were more often diagnosed due to pain than benign ones (30.2% vs. 12.5%, *p* < 0.001). Mixed hormonal activity was also more common in malignant tumors than in benign lesions (20% vs. 1.5%, *p* < 0.001) (**Table 2**).

**Table 2:** Comparison of benign and malignant large adrenal tumors *.

Variables	Benign *n* = 136	Malignant *n* = 63	*P*
Age (*y*)			0.134
Median (Min.–max.)	60.5; (20–86)	57; (18–89)	
Sex: female, *n* (%)	83 (61)	38 (60.3)	0.924
Tumor diameter (mm), *n* (%)			**< 0.001**
Median ±SD Min.–max.	47.0 ± 19.4 (40–150)	75.5 ± 45.2 (40–241)	
Tumor density (HU)			**< 0.001**
Median ± SD Min.–max. Available in *n* (%)	4.0 ± 27.3 (−87–60) 136 (100)	33.0 ± 12.2 (15–90) 60 (95.2)	
Hormonal activity			**< 0.001**
No hormonal excess Hormonal excess Cortisol excess CS MACS Hyperaldosteronism Androgen excess Mixed Metanephrine excess Available in *n* (%)	69 (50.7) 67 (49.3) 65 (47.8) 2 (1.5) 63 (46.3) 0 0 2 (1.5) 0 136 (100)	28 (46.7) 32 (53.3) 17 (28.3) 9 (15.0) 8 (13.3) 0 4 (6.7) 12 (20) 0 60 (95.2)	
Management, *n* (%)			**0.001**
Conservative Surgical	78 (57.4) 58 (42.6)	20 (31.8) 43 (68.2)	
Mode of discovery, *n* (%)			**< 0.001**
Incidental Hormone excess Pain Other	108 (79.4) 0 17 (12.5) 11 (8.1)	23 (36.5) 10 (15.9) 19 (30.2) 11 (17.5)	
Location of adrenal tumor, *n* (%)			0.466
Right Left Bilateral	60 (44.1) 62 (45.6) 14 (10.3)	29 (46.0) 31 (49.2) 3 (4.8)	

PHEO, pheochromocytoma; ACA, adrenocortical adenoma; OB, other benign lesion; ACC, adrenocortical carcinoma; OM, other malignant lesion; n, number; SD, standard deviation; HU, Hounsfield units; CS, Cushing syndrome; MACS, mild autonomous cortisol secretion.

*Biochemically proven pheochromocytomas were excluded.

Bold values indicate statistically significant differences (p < 0.05).

### 3.3 Adrenalectomy

A total of 122 patients underwent adrenalectomy, and 35% of these tumors were malignant. Among resected lesions, 79 (64.7%) exceeded 5 cm in size, of which 36 (45.6%) were malignant. Tumors larger than 10 cm were identified in 24 patients (19.7%); 17 of these lesions (70.8%) were malignant. The majority (88.2%) of malignant tumors larger than 10 cm were adrenocortical carcinomas (ACC), while the remaining 11.8% of cases represented other malignant lesions.

### 3.4 Imaging accuracy in detecting malignancy

Using an unenhanced CT attenuation cutoff of >10 HU, sensitivity for detecting malignancy was 100%, with a specificity of 66.1%. Increasing the cutoff to >20 HU improved specificity to 81.4% while sensitivity decreased to 89.5%. MRI chemical shift imaging demonstrated a sensitivity of 94.4% and a low specificity of 56.8%.

^18^F-FDG-PET/CT showed a sensitivity of 100% for malignancy detection, irrespective of whether the diagnostic criterion was an adrenal-to-liver ratio (ALR) >1 or an SUVmax >5. However, specificity was higher when SUVmax >5 was used compared to ALR >1 (100% vs. 82.1%) (**Table 3**).

**Table 3:** Accuracy of unenhanced CT attenuation, MRI, and FDG PET/CT in detecting malignancy *.

Imaging modalities	*N*	Sensitivity	Specificity	PPV	NPV
Unenhanced CT	*n* = 156				
- HU > 10 - HU > 20		100% 89.5%	66.1% 81.4%	48.7% 60.7%	100% 96.0%
MRI	*n* = 55				
- loss of signal intensity on out of phase < 16%		94.4%	56.8%	51.5%	95.5%
^18^F-PET/CT	*n* = 50				
- ALR > 1 - SUV_max_ ≥ 5		100% 100%	82.1% 100%	61.1% 100%	100% 100%

ALR, adrenal liver ratio; FDG PET/CT, ^18^F-fluorodeoxyglucose positron emission tomography-computed tomography; HU, Hounsfield units; MRI, magnetic resonance imaging; n, number; NPV, negative predictive value; PPV, positive predictive value; SUV, standardized uptake value.

*Biochemically proven pheochromocytomas were excluded.

Bold values indicate statistically significant differences (p < 0.05).

### 3.5 Follow-up

Eighty-seven adrenal tumors were under follow-up. The majority were initially classified as adenomas—59.8% (*n* = 52), followed by myelolipomas—21.8% (*n* = 19), indeterminate masses—11.5% (*n* = 10), and cysts—6.9% (*n* = 6). The mean follow-up time was 30.1 months (range: 6–136) (**Table 4**).

**Table 4:** Prevalence of significant growth, changes in hormonal activity, adrenalectomies, and malignancy in patients under follow-up.

Variables	ACAs *n* = 52 (59.8%)	Myelolipomas *n* = 19 (21.8%)	Cysts *n* = 6 (6.9%)	Indeterminate masses *n* = 10 (11.5%)	Total *n* = 87 (100%)
Follow-up (months)	28.3 (6–163)	34.6 (6–122)	25.7 (8–37)	33.7 (6–79)	30.1 (6–136)
Significant growth*	0 (0%)	3 (15.9%)	0 (0%)	1 (10%)	4 (4.6%)
- mean mm/year		10.8		10.6	
Changes in hormonal activity	5 (13.2%)	0 (0%)	0 (0%)	0 (0%)	5 (8.3%)
- NFAT to MACS	4 (10.5%)				4 (6.7%)
- MACS to NFAT	1 (2.6%)				1 (1.7%)
- NFAT to CS	0				0
- MACS to CS	0				0
[available in no. (%)]	38 (73.1%)	14 (73.7%)	3 (50%)	5 (50%)	60 (69.0%)
Adrenalectomy	6 (11.5%)	0 (0%)	0 (0%)	0 (0%)	6 (6.9%)
Indication to adrenalectomy					
- imaging characteristics - MACS - both	0 (0%) 4 (7.7%) 2 (3.8%)				0 (0%) 4 (4.6%) 2 (2.3%)
Histopathology					
- adenomas - ACC	5 (9.6%) 1 (1.9%)				5 (5.7%) 1 (1.2%)

ACAs, adrenocortical adenomas; NFAT, nonfunctioning adrenocortical tumor; MACS, mild autonomous cortisol secretion; CS, Cushing syndrome.

*More than >20% in maximum diameter in 6–12 months follow-up.

In the adenoma group (*n* = 52), the mean follow-up duration was 28.3 months (range: 6–163). Hormonal reassessment was performed in 38 patients (73.1%). No increase in tumor diameter exceeding 20% per year was observed. Changes in hormonal activity occurred in five patients (13.2%); in four of them with initially hormonal nonfunctioning adenomas, MACS was diagnosed. One patient with baseline MACS demonstrated normal cortisol suppression on the 1-mg dexamethasone suppression test during follow-up. None of these patients developed clinical signs of hypercortisolism or required surgery.

During follow-up, six tumors initially diagnosed as adenomas were referred for adrenalectomy. In four cases, surgery was performed due to hormonal activity. All four patients had MACS at baseline but were initially managed conservatively; subsequent changes in management reflected patient preference and evolving recommendations for MACS. Histopathology confirmed adrenocortical adenoma in all four cases.

In one additional patient, surgery was indicated due to changes in imaging characteristics, including increasing heterogeneity and slight tumor growth (5 mm over 44 months), accompanied by the development of MACS. Imaging demonstrated an absolute washout of 32%, ALR >1 on ^18^F-FDG PET/CT, and SUVmax of 4. Histopathology revealed an adrenocortical adenoma with degenerative changes.

**Figure 2:**
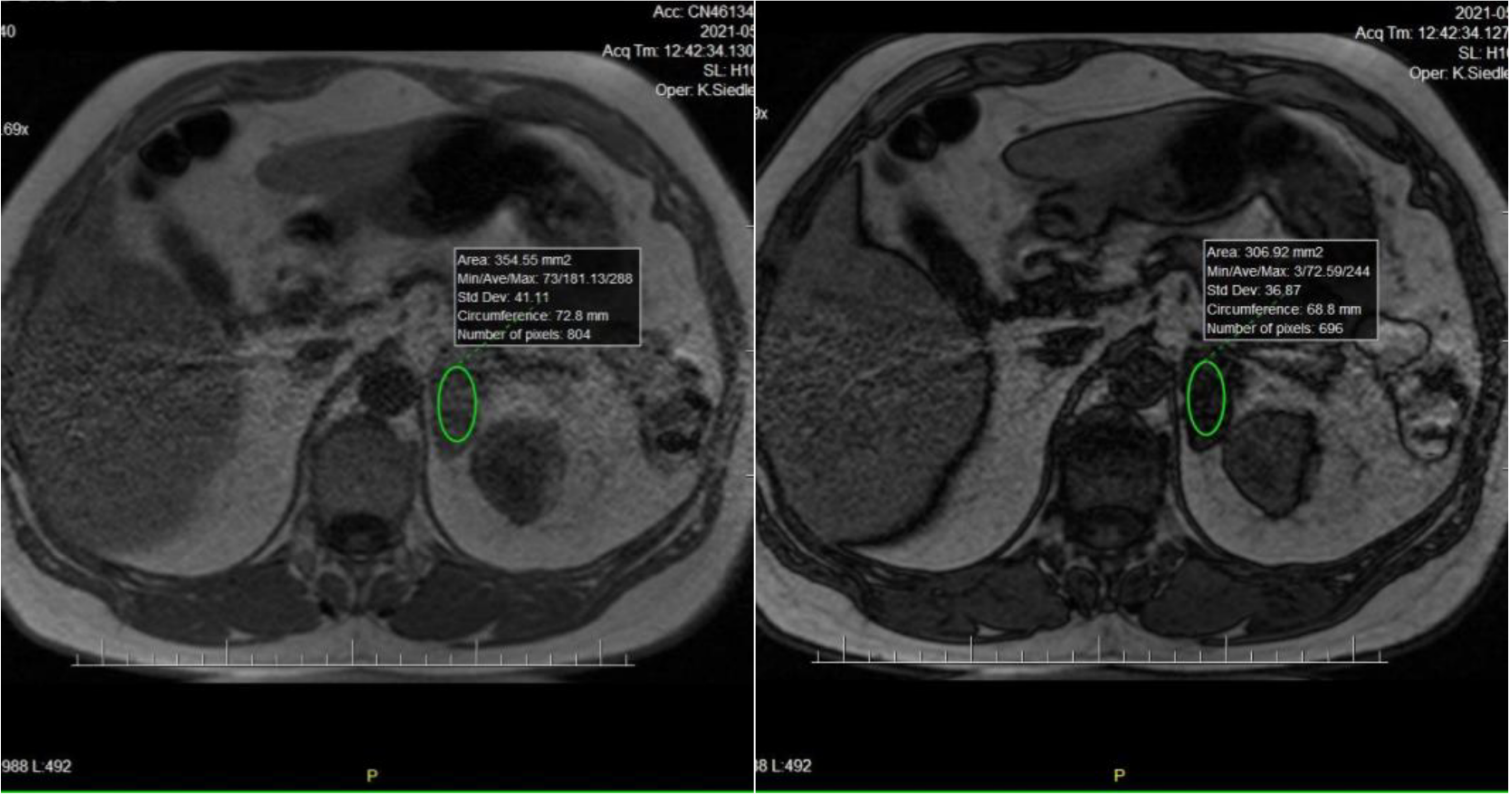
Adrenocortical carcinoma demonstrating signal loss on out-of-phase chemical shift MRI.

In the remaining patient, the lesion initially classified as a non-functioning lipid-poor adenoma showed a marked increase in size, but <20% per year (11 mm per 17 months, mean 7 mm and 18% per year), and additionally MACS was diagnosed, prompting adrenalectomy. Histopathology revealed adrenocortical carcinoma. This female patient had been under observation for 36 months. The left adrenal tumor was incidentally detected on MRI of the spine, followed by contrast-enhanced CT demonstrating a heterogeneous lesion measuring 40 × 25 × 38 mm, with a density of 17 HU on unenhanced CT, 80 HU in the venous phase, and 34 HU in the delayed phase. Relative and absolute contrast washout values were 57% and 73%, respectively. After two years, MRI showed minimal growth (41 × 25 × 40 mm) and 60% signal loss on out-of-phase images on chemical shift analysis (**Figure 2**). At that moment, MACS was diagnosed. Three years after the initial diagnosis, follow-up unenhanced CT revealed further enlargement to 44 × 25 × 51 mm, with a mean attenuation of 12 HU. Due to tumor growth and MACS, adrenalectomy was performed. Histopathology confirmed high-grade ACC (Weiss score 6, Ki-67 >40%), with an adjacent lesion consistent with adrenocortical hyperplasia or adenoma.

In the myelolipoma group, the mean follow-up was 34.6 months (range: 6–122). Significant growth was observed in three tumors (46 mm/31 months, 28 mm/27 months, 31 mm/59 months) including one case complicated by symptomatic intratumoral hemorrhage. None of these patients underwent adrenalectomy.

In the cyst group, the mean follow-up time was 25.7 months (range: 8–37), with no significant changes in size or hormonal activity. Similarly, no hormonal changes were observed in patients with myelolipomas.

Among indeterminate masses, the mean follow-up duration was 33.7 months (6–79). Tumor enlargement was observed in one patient with known extra-adrenal malignancy (urothelial carcinoma of the bladder). Due to the patient’s poor general condition, adrenalectomy was not performed.

## 4 Discussion

LATs are defined as lesions larger than 4–6 cm, with a prevalence ranging from 5.8% to 31% of all lesions, depending on the population studied (5, 29). In this study, tumors ≥4 cm accounted for 18.3% of all lesions. A similar prevalence of LATs (17%) was shown in a retrospective study on 4,085 patients (8). A significantly lower percentage of LATs was reported by A. Ebbehoj in a population-based study, where lesions >4 cm constituted only 5.8% (5). Previous studies reported a higher frequency of LATs, most likely due to limited availability and lower quality of imaging modalities (4, 9, 10).

Based on the current body of evidence, LATs are more frequently malignant (31%–34%) compared to adrenal tumors overall, where the estimated prevalence of malignancy ranges from 3.4% to 11% (6). Nevertheless, the majority of tumors >4 cm are benign (5, 8). The present study provides comparable results, with the prevalence of malignant lesions among LATs reaching 28.7%, while the rate of ACC was 17.3%, which is higher than reported in the largest analysis of LATs (8). Among tumors >5 cm undergoing adrenalectomy, the rate of malignant lesions was higher at 37.4%, and in tumors >10 cm it reached 80.9% (29). In our study, the prevalence of malignancy among patients who underwent adrenalectomy was 45.6% among adrenal tumors larger than 5 cm and 70.8% in tumors exceeding 10 cm. Notably, malignant tumors greater than 10 cm were predominantly adrenocortical carcinomas, accounting for 88.2% of cases.

Non-incidental diagnosis, older age, male sex, larger tumor mass, and indeterminate imaging phenotype are reported as risk factors for malignancy (5, 8). In this study, benign lesions were more often diagnosed incidentally compared to malignant ones (79.4% vs. 36.5%), while in malignant tumors, imaging was more often prompted by pain (30% vs. 12.5%).

The majority of adrenal tumors are non-secreting adenomas (6, 30). In this study, the most common diagnosis was benign adrenocortical adenoma (34.6%), which is in line with the largest study analyzing patients with LATs, which found a 31% incidence of ACA (8). However, in our cohort, as many as 75% of ACA secreted glucocorticoids, which was higher than in the already cited study on LATs, where 46% of adenomas secreted cortisol. When analyzing all the etiologies, almost half of the tumors in our cohort were hormonally inactive (44.7%), and over one-third secreted corticosteroids. CS was diagnosed in 5.1% of patients, including 4.1% with ACC and 1% with ACA. In the already cited study by Iñiguez-Ariza et al., the incidence of overt hypercortisolism among patients with large ACAs was 5% (8). Based on the study by Ebbehøj et al., cortisol-secreting activity is less common in LATs than in adrenal tumors overall, with CS observed in only 0.4% of individuals and MACS in 6.9% (5).

Primary hyperaldosteronism (PA) has been reported in 2%–4% of both small and large tumors (8, 27, 29). In this study, aldosterone secretion was combined with excessive cortisol secretion; no case of isolated aldosterone hypersecretion was found. It may have been caused by the small number of patients referred to our center with suspected PA.

PHEO accounted for 9.6% of etiologies in our cohort, which is lower than in the cited study reporting 22% of LATs due to PHEO (8). In 100% of histologically confirmed PHEO, elevated catecholamine secretion in plasma or 24h urine collection was found preoperatively. This aligns with the high sensitivity of the LC-MS/MS method (90%–95%) in detecting pheochromocytoma (1, 6, 28, 31).

In the present study, all malignant lesions demonstrated unenhanced CT attenuation values >10 HU, yielding a sensitivity of 100%. However, nearly one-third of benign lesions also had a density >10 HU (specificity 66.1%). Increasing the cutoff to >20 HU improved specificity (81.4%), but sensitivity was lower (89.5%). This is in line with numerous studies confirming the high sensitivity (93%–100%) of >10 HU in detecting malignancies, with specificity around 70% (5, 12, 17). In LATs, sensitivity for ≥10 HU remains high (100%), but specificity is low. Raising the threshold to ≥20 HU significantly improves specificity (46% vs. 71%) (8), which was also observed in our study.

As suggested by the current ESE guideline, adrenal tumors with density ≥10 HU may require additional imaging. MRI with chemical shift imaging has a sensitivity around 86%–90% and a specificity of 85% for malignancy detection (12). A recent systematic review and meta-analysis showed 94% sensitivity and 95% specificity for qualitative chemical shift imaging in detecting ACA (32). In our study, MRI had 94.4% sensitivity and 56.8% specificity.

^18^F-FDG PET/CT represents another valuable modality for differentiating adrenal lesions. A recent meta-analysis reported 91% sensitivity and 91% specificity (33). False positives may occur in PHEO and false negatives in renal cancer metastases, well-differentiated neuroendocrine tumors, necrotic lesions, and small malignancies. ALR > 1.8 has 86% sensitivity and specificity; SUVmax >4.5 has 89% specificity and 76% sensitivity (17). In this study, ^18^F-FDG PET/CT demonstrated 100% sensitivity using both ALR >1 and SUVmax >4.5, whereas specificity was higher for SUVmax >4.5 than for ALR >1 (100% vs. 82.05%). Higher specificity of ^18^F-FDG PET/CT compared to MRI may result from more common degenerative changes (necrosis, calcifications, hemorrhage) in LATs, which do not demonstrate signal loss on out-of-phase images.

Data regarding the natural history of LATs remain limited. In a recent systematic review and meta-analysis of hormonally inactive tumors and those with autonomous cortisol secretion (ACS), the average tumor growth was 2 mm over 52.8 months. Adrenocortical adenomas with a baseline size >2.5 cm had a lower growth potential compared to lesions <2.5 cm. Clinically significant growth (≥10 mm) occurred in only 2.5% of patients, and no cases of adrenocortical carcinoma (ACC) were observed. The development of overt clinical hormonal activity was extremely unlikely (<0.1%) among patients with hormonally inactive lesions or MACS, and only 4.3% of patients with hormonally inactive adrenal tumors developed autonomous cortisol secretion (34). In our study the risk of developing MACS among non-functioning lesions was 6.7%. No progression to overt Cushing’s syndrome was observed among non-functioning adrenal tumors, nor was there any progression from MACS to CS, which is in line with the current knowledge. In a previous meta-analysis, the risk of malignancy was estimated at 0.2%, the risk of developing ACS at 0.3%, and overt Cushing’s syndrome (CS) at 0.3%.

The current guidelines have made a shift in the paradigm concerning further monitoring of adrenal adenomas larger than 4 cm. The guidelines from 2016 indicated no need for further imaging for typical adenomas <4 cm and suggested an individualized approach (including surgery) for patients with tumors >4 cm (18). In contrast, the current guidelines have removed the cutoff of 4 cm based on the data from observations in 1,197 patients with tumors ≥4 cm (8, 35).

In our cohort, significant tumor growth (>20% per year) was observed in four lesions (4.6%), including three myelolipomas and one indeterminate mass in patient with extra-adrenal malignancy. Myelolipomas are benign, usually big tumors. Despite their high growth potential, myelolipomas remain benign lesions. Acute hemorrhage may occasionally occur in large myelolipomas, resulting in rapid enlargement of the lesion (36). In our study, significant tumor enlargement was observed in 15.9% of myelolipomas; the mean growth of these lesions was 10.8 mm/year. However, an indeterminate lesion in a patient with an extra-adrenal malignancy always raises suspicion of metastasis. In our patient, a surgical intervention was not performed due to the patient’s poor general condition. In our study there was one case in which a lesion initially classified as benign was later diagnosed as adrenocortical carcinoma. The detection of malignancy during follow-up is more likely related to the initial misclassification of malignant tumors as benign than to malignant transformation of previously benign lesions. In two meta-analyses the risk of developing malignancy was assessed at 0%–0.2%. The reported cases of “malignant transformation” were likely malignant lesions that had been incorrectly classified as benign at the time of initial evaluation (34, 37).

### 4.1 Strength and limitations

A limitation of this study is its retrospective design, which resulted in incomplete availability of patient data, as well as by the relatively high number of patients lost to follow-up. However, all patients were evaluated by the same team of experts, ensuring a standardized approach to patient management. Additionally, adrenal vein sampling is not performed at our center, which limits referrals of patients with suspected primary aldosteronism and likely leads to an underestimation of PA prevalence within our cohort. Conversely, the tertiary referral profile of our center may contribute to an overestimation of the prevalence of ACC. Nevertheless, the relatively short duration of follow-up represents an additional limitation of this study.

### 4.2 Conclusion

LATs are most commonly diagnosed incidentally. Although the risk of malignancy is higher than in lesions smaller than 4 cm, the vast majority of LATs are benign; in our study, 71.4% of tumors were non-malignant. The diagnostic accuracy of imaging studies for detecting malignancy in LATs is comparable to that observed in smaller lesions; therefore, tumor size alone should not be considered an independent indication for surgery. All malignant tumors and pheochromocytomas demonstrated attenuation values >10 HU on unenhanced CT, confirming that this is an excellent parameter for differentiating adrenal lesions, including LATs. Considering that approximately one-third of LATs are malignant, a comprehensive assessment of these lesions is essential. For indeterminate lesions, additional imaging is commonly required, with FDG-PET/CT demonstrating the highest sensitivity and specificity for malignancy detection compared to other imaging modalities. The development of CS in non-functioning tumors is extremely rare. Conservative management may be safe in appropriately selected patients, and follow-up imaging is not required in LATs meeting certain benign imaging criteria, such as homogeneity and attenuation <10 HU. All other lesions require follow-up, preferably managed by a multidisciplinary team.

## Data Availability

The raw data supporting the conclusions of this article will be made available by the authors, without undue reservation.
